# Address at the Commencement of the New York College of Veterinary Surgeons

**Published:** 1895-04

**Authors:** James Law


### THE JOURNAL

OF

### COMPARATIVE MEDICINE AND VETERINARY ARCHIVES.

VOL. XVI. *APRIL*, 1895. NO. 4.

### ADDRESS AT THE COMMENCEMENT OF THE NEW YORK COLLEGE OF VETERINARY SURGEONS.

BY PROFESSOR JAMES LAW.

TO-DAY you are welcomed into the ranks of a profession of honorable aim and of nearly boundless possibilities. Hitherto you have been, as it were, in the parental nest, tended with unremitting care by a most assiduous *alma mater*, who fed you with the pabulum best adapted to your growth and evolution, and lovingly watched over your development of scientifie muscle and the spread of your professional pinion. Now you are adjudged capable of independent effort, and are sent forth, alone, to face alternately the generous sunshine and the winter's blast. In this vital crisis of your life I have the privilege and honor of offering you a few words of encouragement, and perhaps of suggestion, which may be of some help in this important transition period.

You have been individually scrutinized, and the jury of award has pronounced you capable of alone facing the storms and sunshine of the world—capable of entering on personal work for the welfare of the race.

To a class which has just passed through the trying curriculum of a college course I need not say that the diploma just awarded you is not a guarantee of perfection. You feel more intensely than I can impress upon you that you have only had time to grasp the general principles of the science, and how much remains to be learned before you can administer to the sick and suffering with clear intelligence and conscious power. The days are past when the barber surgeon exhausted remedial applications by the letting of blood, or when the farrier, with fleam and drenching horn, administered to the highest needs of the lower animals. Yet these days are only a short remove from the present, and those of us who have reached middle life can recall doctrines and practices which might well be conceived of as lingering shadows of those benighted times.

Formerly all occult diseases of the horse's forelimb were referred to the shoulder or chest, until early in the present century James Turner let in the light by demonstrating the frequency of lesions of the pedal sesamoid pulley. I have myself been taught that that contagious scourge—the rinderpest—was simply the result of unwholesome fibrous food and of its impaction in the omasum. I was further instructed that rabies was due to atmospheric conditions, and that glanders could be caused by overwork, close confinement, and under-feeding. Still more recently—and as it seems but yesterday—tuberculosis was held to be generated by impure air, and some belated beings are still groping about in the dense fogs of this opinion.

In those days a brilliant theory did duty for a sound demonstration, a plausible presumption for attested fact. But our lot has fallen on a new era. The medical idol of yesterday falls under the sledge-hammer of the iconoclast of to-day—the time-honored doctrine is treated with suspicion and skepticism, and the question is no longer "what say the authorities?" but "what is the truth?" The doctrines founded on *a priori* reasoning are at best only tolerated, while those that rest on well-observed facts and otherwise unexplainable experiment are alone accorded the meed of stability. We have learned to doubt and analyze, to question nature as to her secret processes, and to hold no doctrine, however hoary and respectable, as more certain than the evidence on which it rests. As we take the evidence of the eye, of the microscope, of the spectroscope and polariscope, of the test-tube and crucible, of every sense in its turn, of the thermometer and electric machine, of reflexes and interdependent functions, of all the aids that physics can furnish, we feel that we come nearer and nearer to the absolute truth, but the complexity of life processes increasingly warn us how much there is beyond our clearest vision, and at the same time encourage us with the thought of how many new worlds remain to be discovered and how many new fields to be conquered by the earnest, well equipped, and clearheaded worker.

Nor is the old knowledge to be held as in any sense obsolete. Our new discoveries in the regions of microbiology and disease-germs do not altogether exclude many unhygienic influences which have long been recognized as modifying the severity and spread of epizootics. We know, for example, that tuberculosis and glanders are respectively due to their particular bacilli, without which neither disease is possible. But we do not forget that impure air, insufficient food, and debility are still most potent accessory causes of both these affections. Lack of sunshine and ozone, lack of muscular exercise and vigor, and the presence of a hereditary predisposition are no less conducive to tuberculosis to-day than they have been in time past. If we have discovered a cause which is more essential, it does not in any sense abolish those other and accessory causes which were so patent in the estimation of the many careful observers of other days. It is true that we can explain many of these secondary causes through the known action of the inimical conditions on the bacteria and their products, and that our knowledge is thereby founded on a more substantial basis; but perhaps this should lead us to venerate rather than despise the great minds of the past which connected the primary accessory cause with the final pathological result, though unable to see the intervening links in the chain of causation.

Another important thought is that the new science of bacteriological pathology has opened a field of investigation into which we have as yet done little more than enter. We have learned much, it is true, of the morphology and life-history of most of the more prominent disease-germs; we know something of how they comport themselves in different fields of culture, in different genera of animals, and in the same germs under varying dietaries and environment, in neutral, alkaline, and acid mixtures, and under other dissimilar conditions of life; we have some information as to their poisonous products, their ptomaines and toxins; we know something of their chemiotactic powers and their susceptibility to the attacks of phagocytes and protective proteids; but we know as yet comparatively little of the interaction upon each other of the ptomaines and toxins or of the non-poisonous products of different bacteria. It is only in a few isolated cases that we can predict what will be the probable result of the presence in the same animal system of any two different species of bacteria at once. We cannot tell when the two will mutually act as antidotes of each other and when the convalescence and mutual reaction of their products will produce a deadly poison. Here there remains an almost illimitable field as yet practically unexplored. On the other hand, we know that the leucocytes of the body attacked are stimulated to the production of an increase of defensive proteids, globulins, nucleins, alexins, or antitoxins, and we have learned to solicit the extra production of these both for protective and curative purposes, but we are as yet far from an adequate knowledge of the limits of their availability or usefulness.

Some prominent landmarks stand out boldly in the sea of comparative obscurity:

1. Whenever a disease is self-limiting, so that it either kills its victim or results in a speedy and perfect recovery, with immunity from a second attack, we can, by the introduction of the toxic products of the germ, stimulate to the excessive production of antitoxins, which will confer immunity on the animal so treated.

2. When an animal is already infected with such a disease the antitoxins derived from an already immunized animal may be employed as an accessory curative agent.

Of diseases of this class occurring among our domestic animals may be named rinderpest, lung plague, anthrax, rauschbrand, strangles, canine distemper, rabies, tetanus, and a variety of others.

A number of other contagious diseases are productive of a less certain and durable immunity, and in dealing with these we cannot hope for a corresponding success by this method. A malady which may last for a number of years in the same animal system, like tuberculosis or glanders, or which recurs again and again, like aphthous fever or influenza, cannot be thus treated with any confidence in a permanent immunity. At the same time, this need not forbid the use of antitoxins taken from an immunized animal, as accessory curative agents, or as a temporary, if even only as a partial, prophylactic. Our weakness here is in our inability to stimulate the leucocytes to the *permanent* production of the protective proteids in sufficient amount.

Here, then, we are still confronted with uncertainty and imperfect success, and it remains to be seen whether the future will open up new methods which are as yet obscure and untried.

So, also, in regard to the use of the native antitoxins of insusceptible genera of animals, and so with the extracts of special parenchymatous organs that seem to be more or less inimical to particular pathogenic bacteria. How far these may be used as simple curative agents, and how far they may be produced in the body as preventive agents, it is for the future to show.

I need not follow this attractive subject farther. Its limits are as yet far beyond our ken, and what has been already revealed and the great questions that still remain for solution may well inspire us to enthusiasm in the cause of progress. It requires no prophet to foretell that in the near future, when this field shall have been a little further exploited, potential agencies as yet undreamed of by the average layman will be employed in prophylactics and therapeutics, so as to completely eclipse the most brilliant advances of the past. In the theory of the clear-sighted scientist many of these developments have already assumed definite form, and we may well believe that time and opportunity alone are wanted to insure their practical demonstration.

This is a field in which there must arise a great demand for the help of the comparative pathologist in the near future. Such help is needed in the pathological and experimental laboratory, in the flocks and herds, on our farms and ranges, in the stockyard and abattoir, in the creamery and the market.

An enlightened public sentiment must sooner or later transfer all such duties from the mere political placeholder to the man who has been educated to act with the intelligence and clear insight of the scientist in such matters, when a special sanitary duty will be intrusted only to the sanitarian who is a specialist in that field. This time has not yet been reached, however, and we still see public prints inveighing against the rapacity of the veterinarians, who are supposed to be placehunters when drawing attention to sanitary needs.

But while a rational veterinary sanitary work is still a dream of the future, the idea is more substantial and its realization looms nearer than ever before. There is a gradual awakening of the public mind from the hypnotism which has long oppressed it in the matter of popular government. The people begin to look beyond the squabbles of party and the mere struggle for personal and partisan aggrandizement, and to demand a civil administration, the first object of which shall be the effective service of the people, and not a mere spoil of partisan success. The tide of public political conscience is rising, and the reform of our civil service in the direction of ability and efficiency must be allowed to advance until it shall pervade all departments of our administration. The time is past when it might have been stemmed and turned back, and the man or the party which would seek to check it must submit to be swept away and submerged in its onward rush.

When that tide shall have risen to its full the specialist in veterinary sanitary knowledge will be called on to fill the breach for which he has fitted himself, and to protect the flocks and herds and, indirectly, the community from the dangers that beset them in this direction.

Here I must be borne with, if I seem to leave my subject, to offer one word of political advice. You go forth, not as veterinarians alone, but as citizens of this great Republic. Let your profession and yourselves be honored and respected through the position which you assume as citizens. Never condone a flagrant evil or wrong committed by any political party; never contribute actively or passively to any corrupt exercise of the ballot to any partisan distribution of spoils, or engineering of a job, nor to anything else in short that threatens to oppose or hinder a sound administration for the best welfare of the people. Never in your own hearts allow the thought of personal or party advantage to obscure the principles that all public office is a most sacred public trust. If you should fail in this respect, you ought no longer to complain when all your laboriously acquired knowledge and skill are ignored, when your special fitness for veterinary sanitary work is overlooked, and when as the result of a would-be sanitary movement brought about, perhaps, largely, by your own efforts to educate the community, you see the offices filled by political dependents who have no education, no experience, no such skill as to fit them for discharging the duties assigned them. If by your indorsement of corruption or maladministration you have contributed to foul the political stream at its source, you have no right to blame others if the stream proves to be impure below. If by passive acquiescence or active work you have contributed to undermine the sacredness of a public trust, you must take the natural consequences, even if these should come in the form of the waste of public money in alleged sanitary reforms which become but prostitutions of the name under unfit appointees, to the contempt of sanitary science and the discredit of the veterinary profession and its practitioners. If you aim at a public office with sanitary duties, you must do your best to acquire a thorough efficiency in all that will fit you for the discharge of such duties.

Then when the office seeks *you* you will be prepared to fill it with personal credit and with profit to the public.

Veterinary sanitary science is especially a question of political economy, and those who would officer any veterinary sanitary organization with laymen fall back on the necessity of having business men, financial experts, to direct the undertaking. The same principle is often invoked in the case of medical appointments, and sanitation and philanthropy are alike balked through an administration which is unacquainted with the medical questions involved. It is well, therefore, that in furbishing your weapons for work in this field you should give a thorough study of the economic questions involved, and lay before the people the results of such exhaustive investigation. Veterinary sanitary work is a Christian and humane movement, but it is even more directly and primarily an economic undertaking. You must present it to the public on all sides as a means of protecting and husbanding human health and life, and as a means of cutting off the constant losses in our live stock possessions and of increasing the number and value of our flocks and herds.

In sanitary and preventive medicine there is a very wide field which must open ere long to the veterinary expert. The immense numbers and value of our live stock may well illustrate this. According to the census of 1890, our solipeds and meat-making quadrupeds kept on farms in the United States exceed 160,000,000 head, exclusive of the large numbers kept in cities. What a field this presents for sanitary work in the matters of improvement of general hygiene, in the suppression of contagia, in the restriction and cure of parasitisms, in immunizing and other prophylactic treatment, in meat inspection, and in the doing away with enzootic poisons! It is quite true that as yet the greater part of such work is prospective rather than real. But let the expert show the value of such work and his fitness to undertake it, and little by little his services will be availed of *here* as they have long been to a certain extent on the other side of the Atlantic.

Coming to our own State of New York and to one single contagious disease in which the cities have, if possible, a more direct personal interest than the stock owner—tuberculosis—we find a bovine population of 2,328,015 head, with, according to estimate, 5 per cent. of them tuberculous. This percentage at $20 per head would mean $2,328,000 of money value lost or deteriorated because of one contagion. And so long as we leave this contagion to go on preying on the herds we must look for a constant succession of new cases, so that the losses are continuous and permanent. If now we glance at the human mortality from tuberculosis, amounting to 13 per cent. of all deaths, and to which the disease in animals materially contributes, we find reason enough for an active crusade in expert hands against this plague of our flocks and herds.

But all of you will not aim at this as your life's work, and in veterinary medicine there is of necessity a large amount of specializing in practice. It is not necessary for us, as it is for the physician of man, to map out a single body and assign different organs to different experts. From the great variety of live stock it is open to each of us to specialize on some particular genus in the medicine of which we may strive to excel. According to the location selected by us, indeed, we shall be compelled to devote ourselves to one of the genera more than another. If settled in a city, we become, of necessity, mainly or exclusively physicians of the horse, whilst if in a country dairying region we must give our special attention to the cow. Wherever the wealth of the district is concentrated in a particular genus of live stock there our services will be most in demand, and to fill that demand in the most acceptable and profitable way to our employers we must subordinate our knowledge, skill, and energy.

In the cities you will be called upon to examine horses for soundness, and for no item of your duties should you prepare yourselves more carefully. Woe unto you if you overlook a cataract, a carious tooth, a laryngeal paralysis, a drug-concealed lung disorder, an unsoundness of back or lungs, or any other depreciating disqualification. Let it be once supposed that you have been in collusion with the seller to wrong the buyer, and your prospects for success in that quarter will be seriously impaired. To fill the rôle of examiner for soundness you must be most accomplished in anatomy, physiology, and pathology; and in addition you must be a man of quick observation and just judgment, and one whose character is in every respect above suspicion.

If you are in a stock-raising district, you will require expert knowledge and skill in other directions to which the city veterinarian is a comparative stranger. You should know the differences and relative economic value of different geological formations and soils, the approved modes of culture, the nature composition, and dietetic values at different stages of growth of the various fodder crops; the relation of different foods to health, growth, development, gestation, milk-producing condition; the balancing of rations for the different animals under the different uses to which they are put; the selection of individual animals for breeding, for draught, for carriage, for sport, for dairy, for wool, and for meat products; the relation of live stock to the maintenance of a permanent fertility of the soil, and to the improvement of that which is less fertile; the special hygiene of gestation and suckling, and a host of other points that conduce to the prosperity of your employers and of the nation.

In short, you must identify yourself with the advancement of the public weal in all that relates to the live stock industry, and so far as your education can be made conducive to such progress. If you settle down and merely wait for cases of sickness and lameness that you may treat, and operations that you may perform, you will come far short of your duty and opportunity, and you may expect that you will come short also of obtaining the esteem, confidence, and employment that should otherwise be your reward. Wherever a door opens it must be your study to be ready to enter it. If in a sheep district, you should concern yourselves with all that will conduce to improvement in wool and mutton, whether in breeding or dressings, as well as in measures to guard them against epizootics and parasitisms. If in a pork-raising district, your attention will be called to the plagues that now sweep away hogs to the value of scores of millions annually.

You will best approve yourselves true physicians by cultivating your talents and education in the direction of the local demand and by devoting them in the best way possible toward the filling of this demand. It is in this respect of adaptability that education and knowledge tell, and it is here that their exercise is to be recognized and respected. To the mere empiric all phases and stages of a disease which goes under a given name present alike the demand for his one remedy for that disease. In the hands of the physician the remedy will be adapted to the exigencies of the case and the idiosyncrasy of the patient, no matter what may be the name of the malady, and the failure of one remedy will not be the discomfiture of the practitioner, but only the indication for another which may operate unaffected by idiosyncrasy or special condition. So in the general field the uneducated man is helpless for any real good, while he who is well grounded in science can prove himself a valuable adviser and a guide in lifting the whole community into better methods, in stimulating to a new prosperity, and indirectly in laying a foundation for his own future success in the increasing success of his employers.

I do not say that you will find yourselves fitted, without further effort, to fulfil all these duties. Far from it. You have, as it were, served an apprenticeship. You have been taught to use your tools, but when you go forth from your alma mater you must not consider that you are any the less students, nor must sloth nor self-indulgence be allowed to divert your eyes from the height of more perfect knowledge and skill toward which you aspire. In these days of rapid progress in medical science, to stand still is to retrograde, and to retrograde is to sacrifice the vantage-ground on which you started, and to see other younger and less favored men distance you in the race.

If you would be worthy members of your profession, you must never let the student spirit subside. You may pass from youth to age, your hair may whiten and your step lose its firmness, but the spirit of the student must remain with you fresh and green.

> The patriarch pupil must be learning still
> And dying leave his lesson half unlearned.

Thus will your rich experience and treasured knowledge be made subservient to sounder views and better practice, and thus will your counsel become more and more valuable with advancing years.

You have now been intrusted with the tools; you have been given the right to enter the field of labor; but your course must still be like that which you have just completed—a progressive one—every new step based securely on a foundation already laid. As osteology had to precede arthrology and myology; as a knowledge of the macroscopy and microscopy of the tissues had to precede the study of their functions; as a thorough acquaintance with structure and function had to go before the study of pathology, and as etiology and pathology had to prove stepping-stones to therapeutics, so now you have got the means of study and of successful advance by yourselves, and it is for you to put that power to a good and effective use. Much will depend on the soundness of the foundations you have already laid; not less will depend on how you will hereafter build on these foundations. Of this rest assured, that you cannot afford to lose sight of a single phase of the curriculum through which you have just passed. As you pass into active practice you will find the need for one and another of those branches of knowledge which you may have been at times tempted to look upon as unnecessarily abstruse, speculative, or disconnected from your lifework. To keep abreast of science you must be in touch with contemporary literature, and in grasping every new advance you will feel the need of some old branch of your knowledge.

In this comparatively new country, where veterinary medical knowledge has been to a large extent a desideratum, there are many special phases of disease as yet unrecorded, and it is only by the common contributions of all members of the profession that our science can be advanced in this direction.

I wish to say one word as to tact with animals. If any of you have been city boys and little acquainted with the habits of live stock in health, I would advise him to do his best to overcome such disadvantage. Your employer cannot always tell whether your medical counsel or your prescription is right or wrong, but he can always tell whether you are handy or awkward among animals. If you are at ease in dealing with them, it will always be set down to your credit; if you are awkward, it will as certainly detract from the confidence in which you should be held, and you need not be surprised if the quack, with bold effrontery and ready tact in handling animals, should succeed in gaining support in spite of his profound ignorance of disease and his absolutely destructive prescriptions. A knowledge of men and of animals, and tact in dealing with both, are usually as essential to success in practice as is the most profound knowledge of medical science.

Again, in his dealing with animals the veterinarian must be pre-eminently a humane man. A great proportion of our professional duty consists in the relief of pain, and he who aims at this should sedulously cultivate the spirit and habit of humanity. The firm hand and the gentle manner, the "Suaviter in modo et fortiter in re" should be his essential characteristic. I do not say that he should hesitate to inflict a temporary pain for the relief of a continuous or frequent pain. I do not say that he should hesitate to remove from animals useless organs which are sources of constant loss and of much danger and suffering to both man and beast. But I repeat that all his dealing with dumb animals should be qualified and ennobled by humanity. When he can by local or by general anæsthesia obviate acute suffering his patient is entitled to this consideration, and the same consideration is due to the feelings of the owner, whether the subject be a pet dog or a family horse or cow. A tender consideration for the patient is no less important for its reflex action on the practitioner himself. Kindness and consideration for all others in suffering tend to a calm, well-balanced mind, and favors that equipoise and sound judgment which should characterize the accomplished physician. The irritability of a roughly-used patient, on the other hand, conveys itself without fail to the attendant, ruffles his spirit, disturbs his balance, and interferes with the calmness and soundness of his judgment. A shrewd breeder of fine horses said recently that he wanted no one about his stable who did not sleep soundly and well at night. The nervous and irritable man reacts on the high-strung horse; the irritated horse reacts on the irritable man, and both suffer in consequence. The late Mr. Edwin Thorn allowed no employé to speak in a loud voice in the stable, and his horses were models of good temper and gentleness. His men, I need not add, were equally the gainers.

You know how an irritable horse will recognize the touch of the nervous driver as conveyed through the reins and bit; how the cow will withhold her milk from the rude, inconsiderate, or cruel milker, and how the sheep will thrive in the hands of the calm, deliberate shepherd, but will fall off under the care of the merciless driver.

Calmness, temperance, self-control, humanity make for nobility of character, and this is the goal at which we all should aim. This it is that will elevate the man and his profession in the public mind, and this will secure that recognition and status to which an honorable profession is entitled.

It only remains for me to wish you God-speed in your chosen career. Let your course be true, your purposes noble, your lives worthy. Set to yourselves a high aim in your scientific, your professional, and your moral career. Let your names stand out as beacons of worth, of trust, and of character, esteemed by the community, and cherished by your profession and your alma mater.

